# Migration of a Giant Gallstone Through a Therapeutic Cholecystocutaneous Fistula: A Case Report

**DOI:** 10.7759/cureus.110897

**Published:** 2026-06-15

**Authors:** Amaya Daniels, Ferdinand B Mayer, Michael Hutton

**Affiliations:** 1 General Surgery, Buckinghamshire Healthcare NHS Trust, Aylesbury, GBR; 2 General Surgery, Royal Cornwall Hospitals NHS Trust, Truro, GBR

**Keywords:** cholecystocutaneous fistula, cholelithiasis, gallstone migration, giant gallstone, percutaneous cholecystostomy

## Abstract

Percutaneous cholecystostomy tubes (PCTs) are employed in the management of acute cholecystitis in patients unfit for surgery and may be utilised to create a temporary or permanent therapeutic cholecystocutaneous fistula. While spontaneous passage of small gallstones through such fistulae has been described, the migration of a giant gallstone (≥5 cm) has not previously been reported. We present a case involving an 82-year-old woman with a gallstone measuring 65x40 mm that had traversed a cholecystocutaneous fistula and become lodged subcutaneously. The stone was successfully extracted through incision of the fistula tract, resulting in significant resolution of her presenting symptoms. To our knowledge, this is the first reported case of a giant gallstone migrating through a cholecystocutaneous fistula. CT imaging may substantially underestimate the solid content of a peri-fistular collection. Clinical examination findings should therefore guide surgical decision-making when imaging is equivocal. Giant gallstone migration is a rare but clinically significant complication of permanent cholecystocutaneous fistulae, of which surgeons should be cognisant.

## Introduction

Percutaneous cholecystostomy tubes (PCTs) are used in the management of acute cholecystitis in patients whose comorbidities preclude safe general anaesthesia or surgical intervention [1,2]. The procedure involves percutaneous decompression of the gallbladder under ultrasound guidance, with the tube typically serving as a bridge to definitive cholecystectomy once the patient's condition permits [1,2]. However, in patients deemed permanently unfit for surgery, PCT may represent the definitive therapeutic strategy, with the intention of establishing a permanent cholecystocutaneous fistula for ongoing biliary drainage [2,3].

A cholecystocutaneous fistula forms as a consequence of prolonged tube placement, during which an epithelialised tract develops between the gallbladder and the skin surface. Once established, this fistula may persist indefinitely following tube removal (typically after around six weeks), providing continued drainage of bile and preventing recurrent gallbladder distension. The tract can then either spontaneously close once the infection has settled, or might persist if inflammation or obstruction of the gallbladder is recurrent. Definitive treatment, such as cholecystectomy with excision of the fistula tract, can be considered once the patient is deemed fit enough for surgery. The long-term natural history of such fistulae is not well-characterised, but recognised chronic complications include recurrent infection of the drainage tract, persistent biliary discharge causing significant patient discomfort, and chronic pain at the fistula site [2,3]. These risks must be carefully weighed against the potentially life-threatening consequences of untreated gallbladder perforation in patients unsuitable for cholecystectomy.

While passage of gallstones through a cholecystocutaneous fistula has been described, the stones reported are generally small [4]. Conversely, the occurrence of giant gallstones, defined as those measuring ≥5 cm in their largest dimension, is rare [5-8]. Owing to their substantial dimensions, giant gallstones are generally considered an indication for surgical removal, as their size is associated with significant pain and an elevated risk of complications including obstruction, infection, and perforation [2,9]. Giant gallstones migrating through a cholecystocutaneous fistula have not previously been documented.

## Case presentation

An 82-year-old woman presented in April 2022 with a several-week history of worsening pain and purulent discharge around her cholecystostomy fistula site. Two years earlier, she had presented with a contained rupture of the gallbladder secondary to calculous cholecystitis, with perforation contained by adjacent small bowel loops. At that time, she was not deemed fit for cholecystectomy, despite an Eastern Cooperative Oncology Group (ECOG) score of 2, as she was undergoing active treatment for stage IVb endometrial cancer with metastases to the supraclavicular and anterior cervical lymph nodes. While she may have survived the surgery, the concern was that post-operative recovery and wound healing would be impaired in light of her cancer treatment. A percutaneous cholecystostomy tube was therefore inserted under direct ultrasound guidance to establish a cholecystocutaneous fistula for symptom control. The ultrasound report at the time describes several small gallstones as well as the presence of sludge. The tube was removed seven weeks later, and a well-draining fistula had subsequently formed.

A follow-up computed tomography (CT) scan of the abdomen and pelvis, as well as a cholecystostogram performed in 2021, demonstrated a patent fistula and an absence of cholelithiasis, though contrast failed to transit from the gallbladder into the cystic duct, consistent with chronic gallbladder outflow obstruction. The patient had a relatively stable clinical course thereafter until her presentation in April 2022.

On presentation, she was generally well but in significant pain around the fistula site. She was apyrexial with no nausea or vomiting. Examination revealed a round, slightly bulbous fistula site with patches of darkened granulation tissue and a series of subcutaneous collection sites containing purulent fluid. Three small points of seropurulent oozing were identified. The fistula site was firm to palpation, slightly raised, and surrounded by erythematous borders without evidence of spreading cellulitis. Initial differentials included a purulent collection as well as obstruction of the fistula tract by a gallstone or scar tissue.

Investigations

Her initial blood investigations were significant for raised C-reactive protein (CRP), low haemoglobin, raised alkaline phosphatase (ALP), and low albumin (Table 1).

**Table 1:** Laboratory investigations on admission with UK reference ranges CRP: C-reactive protein; ALP: alkaline phosphatase; ALT: alanine aminotransferase; eGFR: estimated glomerular filtration rate

Investigation	Result	Reference Range (UK)
CRP	114 mg/L (↑)	< 5 mg/L
White cell count	6.5 x 10⁹/L	4.0–11.0 x 10⁹/L
Haemoglobin	97 g/L (↓)	120–150 g/L (female)
ALP	194 IU/L (↑)	30–130 IU/L
ALT	10 IU/L	0–49IU/L
Bilirubin	5 µmol/L	0–21 µmol/L
Albumin	25 g/L (↓)	35–50 g/L
eGFR	66 mL/min/1.73m²	≥60 mL/min/1.73m²
Electrolytes	Normal	—

CT imaging revealed a fistular collection measuring 68x34 mm with apparent migration of a gallstone towards the subcutaneous fistulous collection (Figure 1). While there was a focus of increased attenuation measuring around 10x10mm within the fistulous collection, it lacked the well-defined hyperdense appearance typically seen with calcified gallstones. The true size of the gallstone was therefore not readily apparent on CT, as the stone was largely indistinguishable from the surrounding purulent fluid collection. It is noteworthy that the 2021 CT, performed only 12 months prior, had reportedly demonstrated no cholelithiasis; whether the stone was present but undetected at that time or it developed during the interval period remains uncertain.

**Figure 1:**
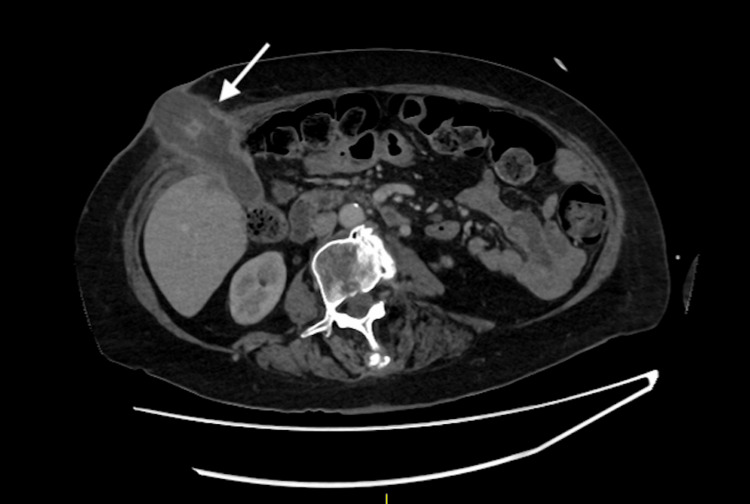
CT imaging demonstrating the fistulous collection (68x34 mm) The gallstone is not clearly distinguishable from the surrounding fluid on cross-sectional imaging, as only a 10 × 10 mm area of increased attenuation is seen. The rest had the same appearance as the surrounding purulent fluid collection.

Management

The patient was taken to the operating room the same day for incision and drainage of the fistula site with the primary goal of exploration and symptom relief. Surgical exploration revealed a giant gallstone measuring 65x40 mm positioned immediately subcutaneously within the fistula tract. The stone was delivered readily once the overlying skin was incised (Figure 2). The fistula tract was irrigated and cleared of purulent material and residual bile, and patency was assessed by direct visualisation and probing. A corrugated drain was placed. The patient's symptoms improved significantly post-operatively. She was managed with a course of empiric intravenous Amoxicillin-Clavulanate in line with local protocol, and regular wound care. Surprisingly, bacterial cultures did not grow any organism. She had an uneventful post-operative recovery and was discharged one week after admission. The drain was removed one month post-operatively, and the wound was allowed to heal by secondary intention.

**Figure 2:**
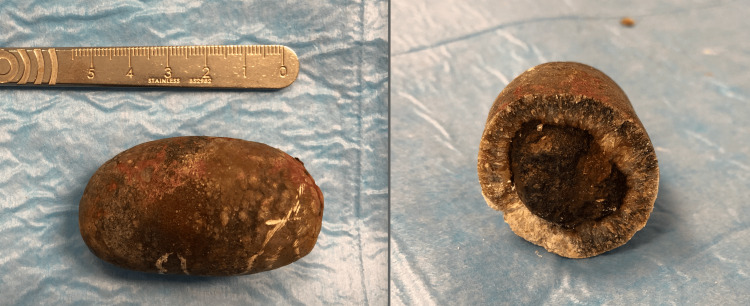
Giant gallstone measuring 65x40 mm removed from the cholecystocutaneous fistula The soft, brittle outer shell was consistent with a cholesterol stone, while the harder core was suggestive of a pigmented component.

Although formal histological analysis was not performed, as it would not have influenced clinical management, macroscopic examination revealed a soft, brittle outer shell consistent with a predominantly cholesterol composition, and a harder inner core suggestive of a pigmented component. This mixed morphology may partially account for the near-isodense appearance of the stone relative to the surrounding purulent collection on CT imaging.

Outcome and follow-up

At the six-month follow-up, the patient was generally well, and the fistula site had healed satisfactorily. Notably, she reported no further symptoms attributable to cholecystitis, supporting the cholecystostomy as a reasonable definitive management strategy in this context. Her clinical course was, however, dominated by significant pain secondary to her metastatic malignancy, and she passed away 12 months following the procedure due to progressive metastatic endometrial cancer.

## Discussion

PCT is a well-established treatment for acute cholecystitis in patients who are not suitable candidates for surgery and is generally well-tolerated [10]. Recognised acute complications include tube obstruction, inadvertent dislodgement, biliary peritonitis, and sepsis [2,10,11]. When PCT is employed with the intention of creating a permanent cholecystocutaneous fistula, chronic complications can include infection of the drainage site, unpleasant patient experience due to ongoing biliary discharge, and persistent pain [11]. These risks must be weighed carefully against the potentially life-threatening consequences of untreated gallbladder perforation in patients unsuitable for cholecystectomy.

To our knowledge, this is the first reported case of a giant gallstone (>5 cm) migrating through a cholecystocutaneous fistula and presents a rare surgical finding. One case report from 2017 describes spontaneous passage of three gallstones through a cholecystocutaneous fistula two years after its formation, with the largest stone measuring 5 cm [4]. A further case describes gallbladder herniation through a cholecystocutaneous fistula with a stone measuring 4x4 cm [12]. The present case, with a stone measuring 65x40 mm exiting through the fistula tract, appears to represent a unique occurrence in the literature.

A clinically important learning point is that giant gallstone migration represents a rare but recognised complication of permanent cholecystocutaneous fistulae, of which operating surgeons should be aware. This complication should be discussed with patients as part of the informed consent process when permanent fistula formation is planned or anticipated. Patients managed in this manner should be monitored for the development or growth of gallstones within the fistula or gallbladder, as unrecognised stones may result in obstruction, infection, and pain requiring surgical intervention.

An important radiological learning point arising from this case is that CT imaging may substantially underestimate the true content of a fistulous collection. In the present case, the 68x34 mm collection seen on CT was largely indistinguishable from the purulent fluid in which the stone was embedded, and the solid gallstone component was not clearly apparent. This has direct implications for pre-operative planning: in patients with a known cholecystocutaneous fistula presenting with peri-fistular swelling and pain, clinicians should maintain a high index of suspicion for an underlying large gallstone even when CT appearances suggest a purely fluid-filled collection. Correlation with clinical examination findings, such as firmness and nodularity at the fistula site, is therefore essential. Where CT appearances remain equivocal, ultrasound may serve as a useful non-invasive adjunct, potentially better delineating the solid components of a peri-fistular collection.

The discordance between the 2021 CT, which demonstrated no cholelithiasis, and the surgical finding of a giant stone one year later warrants consideration. A possible explanation is that the stone was present in 2021 but went undetected. CT has well-documented limitations in gallstone detection, with a reported sensitivity of approximately 75%, largely because cholesterol stones may appear isodense to surrounding bile on cross-sectional imaging [13]. Alternatively, the stone may have formed de novo within the intervening period. While the formation of a stone of this magnitude within 12 months would be unusual, chronic bile stasis within an obstructed gallbladder and an established fistula tract creates conditions conducive to progressive stone formation and growth, and cannot be excluded.

## Conclusions

We present what is, to our knowledge, the first reported case of a giant gallstone (≥5 cm) migrating through a therapeutic cholecystocutaneous fistula, successfully managed by surgical incision and extraction. This case demonstrates that cross-sectional imaging may substantially underestimate the true solid content of a peri-fistular collection, and that clinical examination findings should guide surgical decision-making when imaging appearances are equivocal. Giant gallstone migration represents a rare but clinically significant complication of permanent cholecystocutaneous fistulae, which surgeons should be cognisant of. In patients managed with a permanent therapeutic cholecystocutaneous fistula, recognising stones in the fistula tract is important, as they may precipitate obstruction, infection, and pain, requiring surgical intervention. Prompt surgical management, as demonstrated in this case, can achieve effective symptom relief with a favourable short-term outcome even in patients with significant comorbidity.
